# Curcumin modulates hepatic pyroptosis-autophagy crosstalk induced by aflatoxin B1 via rumen microbiota-blood-liver axis

**DOI:** 10.1186/s40168-026-02396-8

**Published:** 2026-04-20

**Authors:** Tongwei Liu, Shijie Fan, Jiefeng Li, Ting Wang, Jing Zhang, Chuanqi Wang

**Affiliations:** 1 College of Animal Sciences, Jilin University, Changchun, 130062 People’s Republic of China; 2 National Grain Industry Technology Innovation Center (Medicinal Functional Resources Development), Academy of National Food and Strategic Reserves Administration, Beijing, 100037 People’s Republic of China

**Keywords:** Curcumin, Aflatoxin B1, Rumen microbiota, LPS, Hepatic pyroptosis, Autophagy

## Abstract

**Background:**

Aflatoxins, fungal secondary metabolites from Aspergillus species, primarily causes liver and gastrointestinal damage in ruminant. Curcumin, a plant polyphenol, has been shown to possess both anti-inflammatory and antioxidant properties, in addition to regulatory effects on gut microbiota. However, research on curcumin's impact against AFB1 toxicity in ruminants is limited. This study aims to elucidate whether AFB1 induces hepatic pyroptosis and autophagy in ruminants via the rumen microbiota-blood-liver axis and the regulatory role of curcumin. The experimental design involves the administration of AFB1 and curcumin to sheep, followed by a comprehensive observation of alterations in rumen microbiota, barrier function, and the occurrence of hepatic pyroptosis and autophagy, with the aim of elucidating the mechanism of curcumin in ameliorating AFB1-induced liver injury in sheep.

**Results:**

In the experimental setup, 800 mg/kg dry matter (DM) curcumin was administered as a dietary supplement to alleviate the adverse effects of AFB1 (500 μg/kg DM) on the rumen and liver of sheep. AFB1 suppressed NH_3_-N and VFAs production, whereas curcumin improved VFA generation and fermentation efficiency. Curcumin mitigated AFB1-induced rumen barrier impairment by upregulating tight junction proteins (ZO-1, Occludin, Claudin-1) and reducing LPS levels, which was consistent with metagenomic data showing amelioration of microbiota dysbiosis and reduced lysis of Gram-negative bacteria. At hepatic level, curcumin downregulated the principal mediators of the TLR4-NF-κB-NLRP3 signaling pathway (TLR4, p65, and NLRP3), attenuating pyroptosis and reducing serum AST, ALT, and LDH concentrations, while reversing inflammatory infiltration and hepatic cord disruption. Furthermore, curcumin restored autophagic flux by increasing the LC3-II/LC3-I ratio and decreasing p62 accumulation, counteracting AFB1-induced autophagy inhibition.

**Conclusions:**

Curcumin counteracts AFB1-induced rumen-liver axis dysfunction. It works by stabilizing the microbiota, maintaining barrier integrity, and dually regulating pyroptosis and autophagy.

Video Abstract

**Supplementary Information:**

The online version contains supplementary material available at 10.1186/s40168-026-02396-8.

## Introduction

Aflatoxin is a highly toxic secondary fungal metabolite mainly produced by *Aspergillus flavus* and related parasitic fungi, which is widely present in cereal crops and silage [1]. Aflatoxin has received much attention due to its potential threat to agriculture and food safety, and it has been classified as a Class 1 carcinogen [2]. Aflatoxin B1 (AFB1) is one of the most common and highly toxic aflatoxins [3]. Studies have confirmed that AFB1 not only affects animal growth, immunity, and reproductive system, but can also enter the human body through the food chain, causing damage to the liver and kidneys [4, 5]. AFB1-induced oxidative stress and free radical production aggravate oxidative damage and apoptosis of hepatocytes [6]. Significant increases in alanine aminotransferase (ALT) and aspartate aminotransferase (AST) were found in the serum of AFB1-exposed sheep [3]. With a relatively stable rumen microbial community, Dorper × Han crossbred sheep ensure good experimental reproducibility and facilitate the quantification of toxin dose‑response relationships. Moreover, their moderate sensitivity to toxins—avoiding acute death at low doses while consistently exhibiting typical pathological indicators of subchronic toxicity—renders them an ideal model for investigating toxin‑induced injury mechanisms in ruminants.

The hepatic pyroptosis is considered a more critical factor in liver injury. Pyroptosis is a highly inflammatory form of programmed cell death in the innate immune defense system, involved in microbial infections and aseptic inflammation, primarily regulated by NLRPs and caspase-1 [7]. Gasdermin D (GSDMD)-dependent pyroptosis, activated by lipopolysaccharide (LPS) through the non-canonical inflammasome, represents a key driver of inflammatory liver damage. Graveoline can reduce the levels of ALT and AST in liver cells and mouse livers induced by LPS, modulate inflammatory factors, and consequently mitigate liver damage and the infiltration of inflammatory cells [8]. Excessive ROS generated during the metabolism of AFB1 to AFB1-8,9-epoxide (AFBO) by cytochrome P450 enzymes in the liver promotes the binding of NLRP3 inflammasome to pro-Caspase-1 to form a complex, activating Caspase-1 [9]. Activated Caspase-1 cleaves GSDMD protein to form membrane pores, while also cleaving pro-IL-1β and pro-IL-18 to generate active pro-inflammatory cytokines [10]. In liver inflammation resulting from metabolic or toxic damage, autophagy frequently exhibits functional impairments. Specifically, inflammatory signals can inhibit autophagic flux, resulting in the accumulation of proteins such as p62 and mitochondrial dysfunction. This dysfunction activates the NLRP3 inflammasome and triggers the release of damage-related molecular patterns (DAMPs) such as HMGB1, which amplifies the inflammatory response. Conversely, an upregulation of autophagy facilitates the clearance of inflammatory mediators and cellular debris, while also regulating immune cell function, thereby mitigating the propagation of inflammation [11, 12]. Autophagy is an intracellular degradation process characterized by the formation of double-membrane autophagosomes that encapsulate and degrade cytoplasmic components, thereby enabling cells to counteract oxidative stress and maintain cellular homeostasis [13]. Impaired or inhibited autophagy has been shown to disrupt normal cellular functions, leading to apoptosis or cell death [14]. Impaired autophagy flux represents a common pathological mechanism underlying viral, metabolic, and toxic liver injuries, as evidenced by disrupted autophagosome-lysosome fusion in hepatitis B virus (HBV) infection, defective lipid degradation in nonalcoholic fatty liver disease (NAFLD), and compromised clearance of damaged organelles in alcohol-induced hepatotoxicity [15].

Beyond this direct hepatic insult, emerging evidence suggests that AFB1 toxicity may be modulated by extra-hepatic pathways, particularly through interactions with the gut microbiome. Ruminants show greater resistance to mycotoxins, in part due to ruminal microbial biodegradation of toxins [16]. Studies have found the degradation ability of the bacteria *Microbacterium proteolyticum* B204 isolated from cow dung for AFB1 and its potential application value [17]. However, ruminal microbes can degrade only 10% to 50% of AFB1 under in vitro conditions [18], suggesting that a significant portion of AFB1 remains available to exert toxic effects on ruminants. AFB1 affects the fermentation of the gastrointestinal tract and the composition of mucosal flora in goats, damages the tissue structure and function of the gastrointestinal epithelium, and induces excessive production of inflammatory metabolites, thereby causing enterotoxicity [19]. Furthermore, AFB1 changed the composition of the rumen microbiota, and the relative abundance of *Prevotella*, *Ruminococcus*, and *Succinobacter* was significantly reduced, which affected the normal fermentation of the rumen [20]. AFB1 significantly modifies the intestinal microbial community of sheep, leading to reduced microbial diversity and altered community homogeneity in a dose‑dependent manner [21]. Particularly, AFB1 increased the abundance of BF311 genus and the *Firmicutes*/*Bacteroidetes* ratio, and decreased the abundance of *Butyrivibrio* [22].

As a plant-derived polyphenolic compound, curcumin has biological functions such as anti-oxidative stress, balancing intestinal flora, anti-inflammation, and regulating lipid metabolism [23]. Dietary supplementation with curcumin at low concentrations (1–2 mg/kg concentrate) improves growth performance and health status in lambs through enhanced antioxidant and anti-inflammatory effects, whereas higher doses may not confer benefits and could induce adverse responses [24]. Curcumin inhibits the NF-κB/MAPK signaling pathway, promotes the polarization of microglia towards the M2 phenotype, and ultimately alleviates PANoptotic neuronal death caused by cerebral ischemia–reperfusion [25]. Curcumin alleviated AFB1 toxicity by regulating oxidative stress, promoting toxin biotransformation, enhancing immune response, and inhibiting apoptosis [26]. Moreover, curcumin effectively reduced AFB1-induced toxicity by regulating antioxidant and anti-inflammatory responses, cancer and drug metabolism-related molecular pathways [1]. However, its potential to protect ruminants from AFB1 via stabilizing the rumen-liver axis remains largely unexplored.

Therefore, the central question addressed in this study is twofold: First, to what extent does AFB1-induced rumen dysbiosis contribute to the overall hepatic injury beyond the direct chemical toxicity? Second, can dietary curcumin intervention mitigate liver damage by concurrently modulating the rumen environment and hepatic cellular stress pathways? To this end, we investigated the effects of AFB1 and curcumin supplementation on rumen fermentation, microbiota, barrier markers, and key mediators of hepatic pyroptosis and autophagy in sheep. This work aims to provide novel insights into the integrative role of the rumen-liver axis in AFB1 toxicology and to evaluate curcumin as a multi-targeted dietary strategy for mycotoxin mitigation.

## Materials and methods

### Animal ethics

The experimental protocols in this study was performed according to the guidelines of Institutional Animal Care and Use Committee of Jilin University (SY202311011). In addition, all measures were taken to guarantee animal welfare and minimize ethical concerns throughout this trial.

### Animal management and experiment procedure

Eighteen Dorper × Small-tail Han sheep crossed male sheep with similar body weight (29.57 ± 0.91 kg) were randomly assigned into three groups of three replicates each, with two sheep in each replicate. The sheep received basal diet (Ctrl group), basic diet + AFB1 at a dose of 500 μg/kg DM (AFB1 group), basic diet + AFB1 at a dose of 500 μg/kg DM + Curcumin at a dose of 800 mg/kg DM (AFB1-Cur group). The dose of AFB1 was adopted from the study by Cao et al. [27], and that of curcumin was determined based on our previous study in vitro experiment [28]. The AFB1 (A96590, Purity ≥ 98%) was purchased from Shanghai Acmec Biochemical Co., Ltd (Shanghai, China). The whole experimental period contained a 7‐day adaptation to the feeding environment and a 14-day formal trial with dietary AFB1 or curcumin. The sheep were fed the total mixed ration (TMR) diet, and the dietary composition was formulated to meet or exceed the nutrient requirements according to NRC (2007) (Supplemental Table S1). During the whole experiment, all sheep were fed twice daily at 06:00 and 18:00 in free-stall sheep barns. The Ctrl group was fed the basal TMR diet, while the AFB1 group received the TMR diet supplemented with 500 µg/kg DM AFB1. The AFB1-Cur group was fed the TMR diet containing both 500 µg/kg DM AFB1 and 800 mg/kg DM curcumin. The AFB1 was dissolved in methanol and evenly sprayed onto the TMR diet.

### Sample collection

Starting from the formal experiment, the feed intake was recorded daily. the initial and final body weight of each sheep was recorded to calculate the average daily gain (ADG) and average daily feed intake (ADFI). At the end of the formal experiment, 5 mL of blood was collected from the jugular vein of each sheep and immediately centrifuged at 3500 rpm for 15 min. Then, the supernatant was stored at − 80 °C for subsequent detection. All the sheep were stunned, euthanized and dissected after a 12-h fast process. The part of liver was fixed into 4% paraformaldehyde for histopathological observation, and remaining liver and rumen tissues were stored in liquid nitrogen rapidly for further analysis. Meanwhile, the rumen fluid was filtered through four layers of sterile gauze and quickly stored in liquid nitrogen for subsequent rumen microbiota analysis.

### Measurement serum levels of AST, ALT and LDH

Following centrifugation, the serum was collected and subjected to subsequent analyses according to the protocols provided by commercial kits for AST (KTB1420, Abbkine, Wuhan, China), ALT (KTB1410, Abbkine, Wuhan, China), and LDH (KTB1110, Abbkine, Wuhan, China). Absorbance values were subsequently measured using a microplate reader (EON, BioTek, USA), followed by the generation of standard curves to calculate the respective concentrations.

### Lipopolysaccharide (LPS) concentration analysis

The LPS level within rumen fluid, serum and liver tissue of sheep was examined with the chromogenic LAL endotoxin assay kit (C0276S, Beyotime Biotechnology, Shanghai, China) according to the operating instructions. In brief, 10 μL endotoxin detection water, endotoxin standard solution and sample were added to the centrifuge tubes without endotoxin. Then, 10 μL endotoxin detection solution was added, mix well, and incubate at 37 ºC for 9 min in the dark. 10 μL color developer solution was added, mix well, and incubate at 37 ºC for 6 min. 50 μL Buffer A, Buffer B, and Buffer C solution were added in order, mix well and let stand for 5 min. Finally, a microplate reader (EON, BioTek, USA) was used to determine the OD value at a wavelength of 545 nm.

### Measurement serum levels of IL-1β and IL-18

The procedure was executed in the following manner: Initially, standards and serum samples were loaded into designated wells, with 100 μL of diluted standards added to standard wells and 100 μL of serum added to sample wells. Subsequently, 50 μL of detection antibodies for IL-1β or IL-18 were added to each well within 15 min of sample addition. The microplate was then sealed with a film and incubated at room temperature for 2 h. Following this, the plate was washed with Wash Buffer. Subsequently, 100 μL of TMB substrate solution was added to each well, and the plate was resealed and incubated in the dark at room temperature for 30 min. Finally, 100 μL of Stop Solution was added to each well, and the plate was placed in a microplate reader (EON, BioTek, USA), which measured the sample's optical density at 450 nm within 30 min. A standard curve was then plotted to calculate the concentrations of IL-1β (KTE7005, Abbkine, Wuhan, China) and IL-18 (KTE3028, Abbkine, Wuhan, China) in the test samples.

### Histological analysis

The liver of sheep was fixed with 4% paraformaldehyde (BL539A, Biosharp, Hefei, China) and stored overnight in 4 °C before embedding in paraffin via standard dehydration process. After 24 h of fixation, paraffin was cut into 5 μm thick sections, transferred to slides, and then stained sequentially using 2.5% hematoxylin (BL700B-1, Biosharp, Hefei, China) and 0.5% eosin (BL700B-2, Biosharp, Hefei, China). Finally, the sections were viewed and photographed using a light microscope (IX71, OLYMPUS, Japan) with × 200 and × 400 magnification, respectively.

### Fluorescence in situ hybridization (FISH)

The FISH was performed to analyze bacterial translocation in liver. In brief, liver sections were embedded in paraffin and then hybridized with Cy3-labeled EUB338 probes (sequences: EUB338 I 5′-GCTGCCTCCCGTAGGAGT-3′; EUB338 II 5′-GCAGCCACCCGTAGGTGT-3′; EUB338 III 5′-GCTGCCACCCGTAGGTGT-3′). In summary, the prehybridization buffer was applied to the sections and incubated at 37 ℃ for 1 h. Following this, the prehybridization buffer was removed, and hybridization buffer containing the probes (8 ng/μL) was added for overnight incubation at 37 ℃ in a humidified chamber. Subsequent to the hybridization process, the slides were washed to remove unbound probes. This was followed by counterstaining with DAPI (2 μg/mL) for 8 min under conditions that protected the samples from light. Following a thorough rinse, the sections were mounted with anti-fade mounting medium and observed under an upright fluorescence microscope for image acquisition.

### Volatile fatty acids (VFAs) and ammoniacal nitrogen (NH_3_-N) content of rumen fluid

The determination of VFAs was conducted by internal standard method using gas chromatography (7890B, Agilent, USA) with an SH Stillwax quartz capillary column (30 m × 0.25 mm × 0.25 μm). Briefly, the mixed standard solution was prepared by different VFAs (Acetic acid, Propionic acid, Butyric acid, Isobutyric acid, Isovaleric acid) standard samples separately diluted with pure water. Nitrogen was used as the carrier gas, with a carrier gas pressure of 100 kPa and a split ratio of 50:1. Heating program: Hold at 80 ℃ for 1 min, heat up to 170 ℃ at 8 ℃/min, heat up to 220 ℃ at 20 ℃/min, and hold for 4 min. The colorimetric method was used to determine the NH_3_-N content in rumen fluid. A solution was formed by dissolving 0.08 g of sodium ferrocyanide in 100 mL of 14% sodium salicylate solution. Solution B was prepared by mixing 2 mL of sodium hypochlorite solution with 100 mL of 0.3 mol/L sodium hydroxide solution. 10 mL of rumen fluid was centrifuged at 3500 rpm for 10 min, and 2 mL of supernatant was taken and placed in a 15 mL centrifuge tube. Then, 8 mL of 0.2 mol/L hydrochloric acid was added. 0.4 mL of standard solutions of different concentrations were divided into centrifuge tube, and 2 mL of solution A and 2 mL of solution B were added to each tube in sequence. After shaking well, they were allowed to stand for 10 min. Then, a spectrophotometer (UV-1750, Shimadzu, Japan) at 700 nm was used to record the OD value.

### Metagenomics sequencing and analysis of rumen fluid

DNA from the rumen microbiota was isolated and purified using a magnetic bead-based genomic DNA extraction Kit (AU46111, BioTeke Corporation, Beijing, China) according to the operating protocol from the manufacturer. DNA concentration was measured by Qubit 1X dsDNA HS Assay Kit (Q33230, invitrogen). 200 ng of DNA was put into a 0.6 mL low-adsorption centrifuge tube, and water was added to 52 μL. The DNA was interrupted according to the fragment length requirements (generally 200-500 bp), and the interrupted product was recovered by magnetic beads purification using the TruSeq library construction kit. Genomic Library construction was performed with the TruSeq Nano DNA LT Library Preparation Kit (FC-121–4001, Illumina) by end-repair, adapter ligation, index PCR amplification, and purification of fragment products, and then quantified using Qubit 1X dsDNA HS Assay Kits (Q33230, invitrogen). Finally, the library was sequenced at both ends using PE150 sequencing mode according to standard operation of NovaSeq 6000 XP 4-Lane Kit v1.5 (300 cycles) (20,043,131, Illumina) through Illumina Novaseq 6000 platform (LC Bio Technology, Hangzhou, China).

The fastqc was used for quality control of raw data. Bowtie2 (version 2.2.0) was used to align the effective sequences of each sample to each Unigene sequence, calculate the number of alignment reads of each Unigenes in each sample, and filter out Unigenes with less than 2 reads in all samples. The final Unigenes set for subsequent analysis was obtained and the abundance of each Unigenes was calculated. QIIME1 was used to analyze the Alpha diversity index of species at the species level. Beta diversity analysis was performed by calculating Bray–Curtis distance and using PCoA. For the purpose of conducting differential statistical analysis across comparison groups based on species, functional profiles, and unigenes, the Wilcoxon rank-sum test was applied for pairwise comparisons between two groups with biological replicates, while the Kruskal–Wallis test was used for multi-group comparisons. A significance threshold of *P* < 0.05 and |log₂(fold change)|> 1 was adopted to identify differentially abundant features. The abundance patterns of significant species or functional terms across groups were then visualized using grouped boxplots generated in R (version 3.6.0). Furthermore, differentially expressed unigenes were subjected to KEGG and GO enrichment analyses using the R package OmicStudioClassic (version 1.3.17) to elucidate their biological relevance in pathways and molecular functions.

### Metabolomics analysis of rumen fluid

A 20 μL of rumen fluid was extracted with 120 μL of precooled 50% methanol, vortexed for 1 min, and incubated at room temperature for 10 min. The extracts were stored overnight at -20 °C and centrifuged at 4000 g for 20 min before storing at -80 ℃ until subsequent analysis. All chromatographic separations were performed on an ACQUITY UPLC T3 column (100 mm*2.1 mm, 1.8 μm, Waters, Milford, USA) through an UltiMate 3000 UPLC system (Thermo Fisher Scientific, Inc. Bremen, Germany) for reversed-phase separation. Metabolites eluted from the column were detected using a high-resolution tandem mass spectrometer TripleTOF 6600 (SCIEX, Framingham, MA, USA). The raw LC–MS data files were converted into mzXML format and then processed with XCMS, CAMERA and metaX toolbox implemented by R software. The online KEGG and HMDB databases were used to label the metabolites, and the exact molecular mass data (m/z) of the samples were matched with the data in the database. The statistical analysis of the data was mainly completed by R software (version 4.0), the cluster heatmap was drawn by pheatmap, the PCA analysis and the significantly different protein analysis were completed by metaX, the PLSDA analysis was performed by ropls, and the VIP values of each variable were calculated. Correlation analysis was performed by Pearson's correlation coefficient. The three conditions of *p* value < 0.05 obtained by t-test, fold change > 1.2, and VIP calculated by PLSDA analysis simultaneously met to screen out the final significant differential metabolites. Differential enrichment analysis of KEGG Pathway was performed based on hypergeometric test. Functional items with *p* value < 0.05 of statistical test were significantly enriched by differential proteins.

### RNA isolation and RT‑qPCR

The total RNA of liver and rumen tissues was extracted by TRIzol reagent (RE703, Genesand, Beijing, China). RNA concentration and purity were measured using a nanophotometer (NanoDrop 2000, Thermo Fisher Scientific, USA), where RNA samples with an absorbance ratio between 1.8 and 2.1 at 260/280 nm were available for further analysis. RNA reverse transcription reactions were performed using TransScript® Uni All-in-One First-Strand cDNA Synthesis Kit (AT341, TransGenes Biotech, Beijing, China) according to the instructions. Then, the PerfectStart® Green qPCR SuperMix kit (AQ601, TransGenes Biotech, Beijing, China) was used to perform the RT‑qPCR reaction by ABI Prism 7500 system (Applied Biosystems, Foster City, CA, United States). The reaction system consisted of 10 μL 2 × Green qPCR SuperMix, 0.4 μL upstream and downstream primers, and 7.2 μL RNase-Free ddH_2_O. The setting program on the instrument was as follows: predenaturation at 95 °C for 30 s, 39 cycles of denaturation at 95 °C for 5 s and annealing at 60 °C for 34 s. The relative expression of targets gene was calculated using the 2^−ΔΔCt^ method, and β-actin was used as the reference gene. The primer design and synthesis of target genes (Supplemental Table S2) were commissioned by Sangon Bioengineering Co., LTD. (Shanghai, China).

### Western blotting

The rumen and liver tissues were ground to powder and transferred to a 1.5 mL centrifuge tube with 1 ml of RIPA lysate (R0010, Solarbio, Beijing, China), then left at 4 °C for 15 min, centrifuged at 10,000 × g for 5 min to obtain the supernatant for further analysis. The content of total protein was determined by the Enhanced BCA Protein Assay Kit (P0010S, Beyotime Biotechnology, Shanghai, China). A 12.5% SDS-PAGE gel (MA0388, Meilun, Dalian, China) was configured for protein separation according to different molecular weights, then the target protein was transferred to the PVDF membrane (IPVH00010, Millipore, Burlington, USA). At the end of transferring membrane, the PVDF membrane was washed with 1 × TBST solution for 5 min and sealed in 5% skim milk powder for 3 h. The PVDF membrane was placed in primary antibodies that had been diluted according to the manufacturer's instructions overnight at 4 °C. After incubation with the primary antibody, the membrane was washed with 1 × TBST three times for 10 min each and placed in diluted secondary antibody and incubated for 1 h at 25 °C. The details of antibodies used for western blotting were listed in Supplemental Table S3. After completion of incubation, membrane was washed with 1 × TBST three times for 10 min each. Finally, the membrane was tiled on the UVItec Gel imaging system sample plate dropped the ECL reagent and photographed to obtain the relevant bands.

### Statistical analysis

All data are presented as the mean ± standard error of the mean (SEM). Statistical analysis was performed using SPSS software (version 19.0, Armonk, USA). One-way ANOVA followed by Duncan’s post hoc test was used to determine significant differences among groups, with *P* < 0.05 considered statistically significant. Data visualization was conducted using GraphPad Prism (version 8.0, San Diego, USA). The correlation of ruminal microbiota and metabolites was performed using spearman analysis, and *P* < 0.05 was considered significant difference.

## Results

### Effects of curcumin on AFB1-induced changes in growth performance and rumen fermentation parameters of sheep

**Fig. 1:**
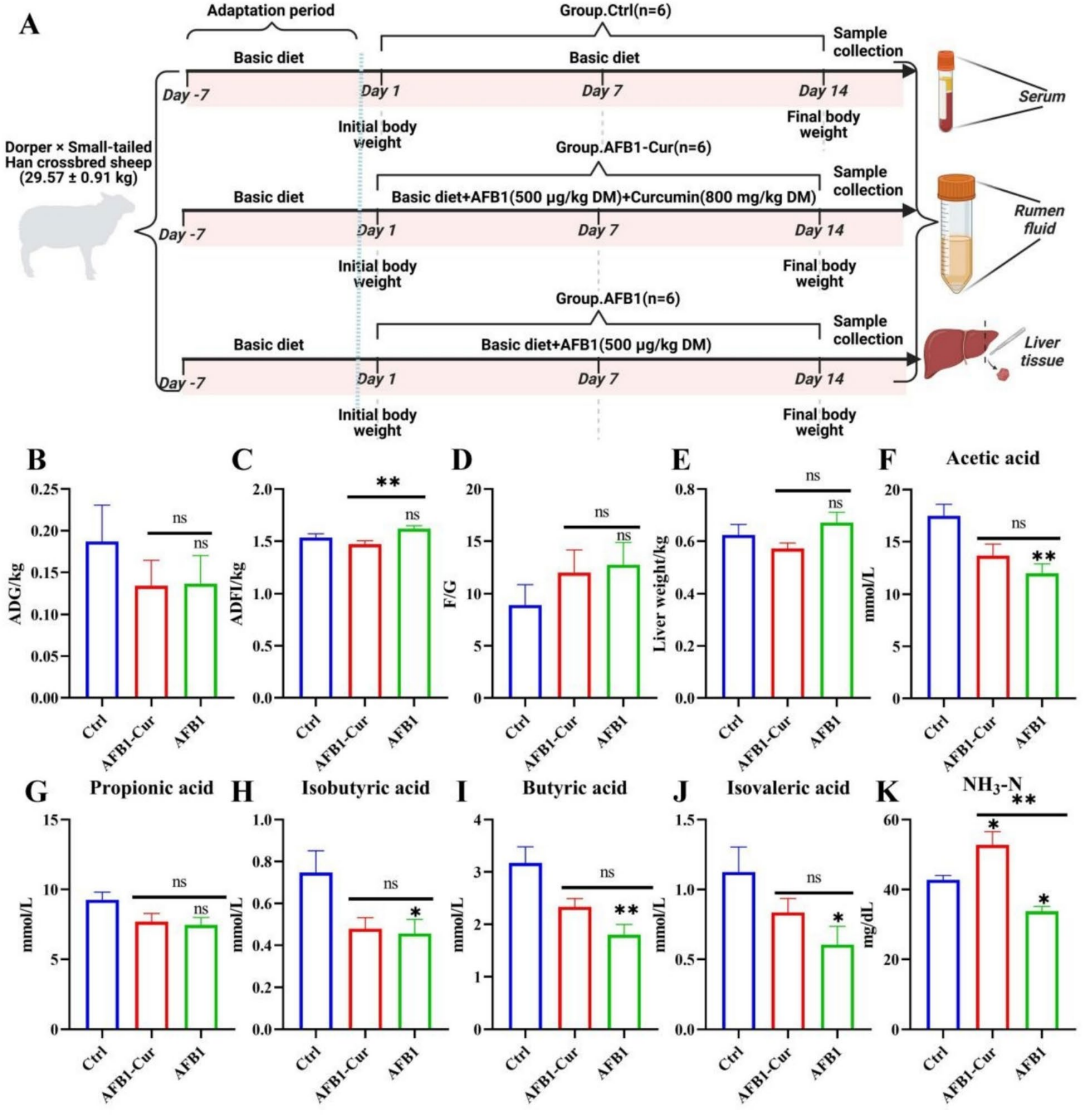
Effects of curcumin on AFB1-induced changes in growth performance and rumen fermentation parameters in sheep. **A** Experimental design. **B** The average daily weight gain (ADG) of sheep. **C** The average daily feed intake (ADFI) of sheep. **D** The ratio of ADFI/ADG. **E** the liver weight of sheep. (**F**-**K**) Fermentation parameter indicators (Acetic acid, propionic acid, isobutyric acid, butyric acid, isovaleric acid, and NH3-N) in rumen fluid. All data are expressed as mean ± SEM, n = 6. * means *P* < 0.05, ** means *P* < 0.01, ns means *P* > 0.05

This work firstly evaluated the effects of AFB1 on growth performance and changes in ruminal fermentation parameters and the regulatory effects of curcumin. As shown in Fig. 1, compared with the AFB1 group, the AFB1-Cur group demonstrated a significantly reduced average daily feed intake (*P* < 0.01). In terms of rumen fermentation, the AFB1 group exhibited significantly lower concentrations of acetic acid, isobutyrate, butyrate, and isovalerate compared with the Ctrl group (*P* < 0.01 or *P* < 0.05). The NH_3_-N level were also significantly decreased in the AFB1 group relative to the Ctrl group (*P* < 0.05), whereas the AFB1-Cur group displayed a marked increase in NH_3_-N compared with the AFB1 group (*P* < 0.01).

### Effects of curcumin on AFB1-induced ruminal microbiota disturbance in sheep

**Fig. 2:**
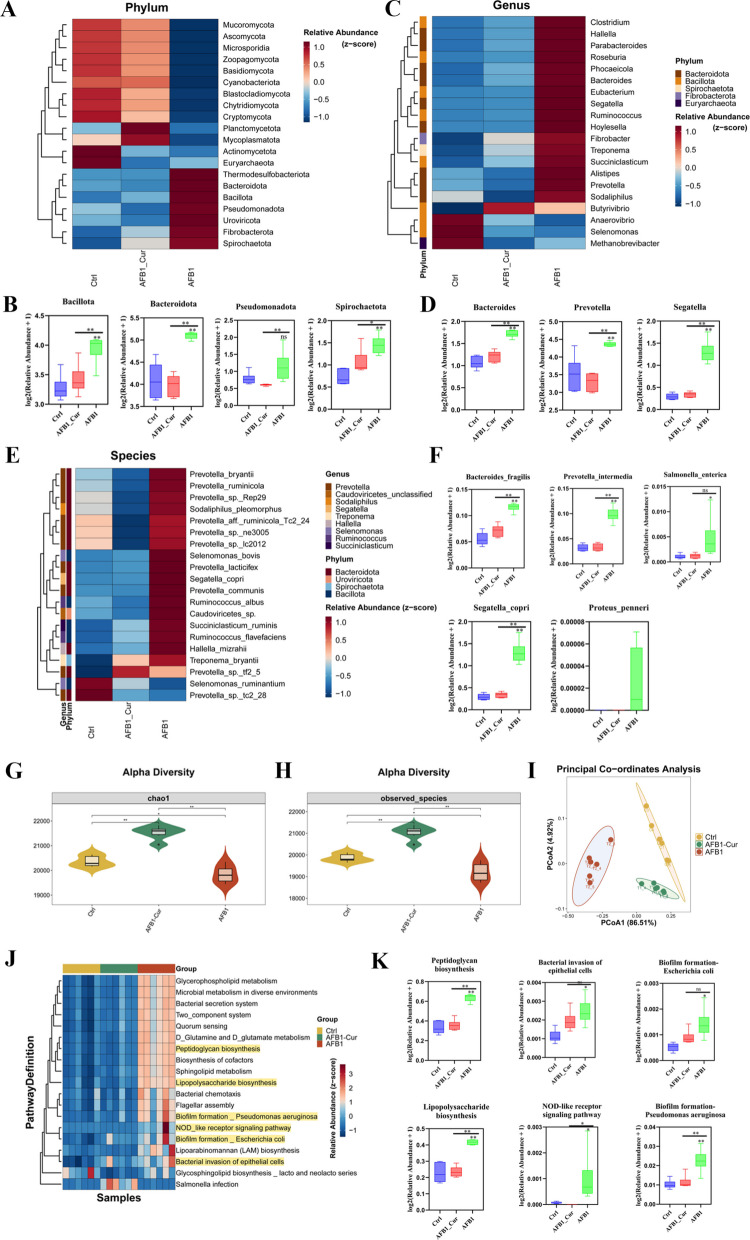
Effects of curcumin on AFB1-induced ruminal microbiota disturbance in sheep. **A**, **C**, **E** Rumen microbial composition at phylum, genus, and species level, respectively (Top 20). **B**, **D**, **F** Differential key microbiota among 3 treatments at phylum, genus, and species level, respectively. **G** Chao1 index of alpha diversity. **H** Observed_species of alpha diversity. **I** Principal coordinates analysis (PCoA) index of beta diversity. **J** KEGG heatmap of Unigenes enrichment pathway in microorganisms with significant differences. **O** Key pathways were enriched in Unigenes among 3 treatments. All data are expressed as mean ± SEM. * means *P* < 0.05, ** means *P* < 0.01

Metagenomic sequencing was used to evaluate the effects of curcumin supplementation on AFB1-induced dysbiosis of the rumen microbiota. Curcumin alleviated AFB1-induced changes in the relative abundance of microbial communities at phylum, genus, and species level, thereby restoring rumen microbial balance (Fig. 2A, C, and E, Supplemental Table S4). The relative abundances of *Bacteroidota*, *Bacillota*, *Spirochaetota*, and *Pseudomonadota* in the AFB1 group was significantly higher than that in the Ctrl and AFB1-Cur groups at phylum level (*P* < 0.05) (Fig. 2D). The relative abundances of *Prevotella*, *Bacteroides*, and *Segatella* in the AFB1 group was significantly higher than that in the Ctrl and AFB1-Cur groups at genus level (*P* < 0.05) (Fig. 2E). The relative abundances of *Segatella copri*, *Bacteroides fragilis*, *Prevotella intermedia*, *Proteus penneri*, and *Salmonella enterica* in the AFB1 group was significantly higher than that in the Ctrl and AFB1-Cur groups at the species level (*P* < 0.05) (Fig. 2F, Supplemental Table S5).

The Chao1 and Observed Species index of ruminal microbiota in the AFB1 group was significantly lower than that in the Ctrl and AFB1-Cur groups (*P* < 0.05) (Fig. 2G and H). The Principal Co-ordinates Analysis (PCoA) revealed significant differences among the 3 treatments (Fig. 2I). KEGG enrichment analysis (Fig. 2J and K, Supplemental Table S6) revealed that, compared with the Ctrl group, the AFB1 group showed a significant upregulation (*P* < 0.05) of microbial unigenes enriched in pathways associated with peptidoglycan biosynthesis, LPS biosynthesis, bacterial invasion of epithelial cells, NOD-like receptor signaling, Pseudomonas aeruginosa biofilm formation, and Escherichia coli biofilm formation. Notably, the AFB1-Cur group showed a marked reversal of these effects, with significant downregulation (*P* < 0.05) in the same pathways compared with the AFB1 group.

### Effect of curcumin on AFB1-induced ruminal microbial metabolic disorders in sheep

**Fig. 3:**
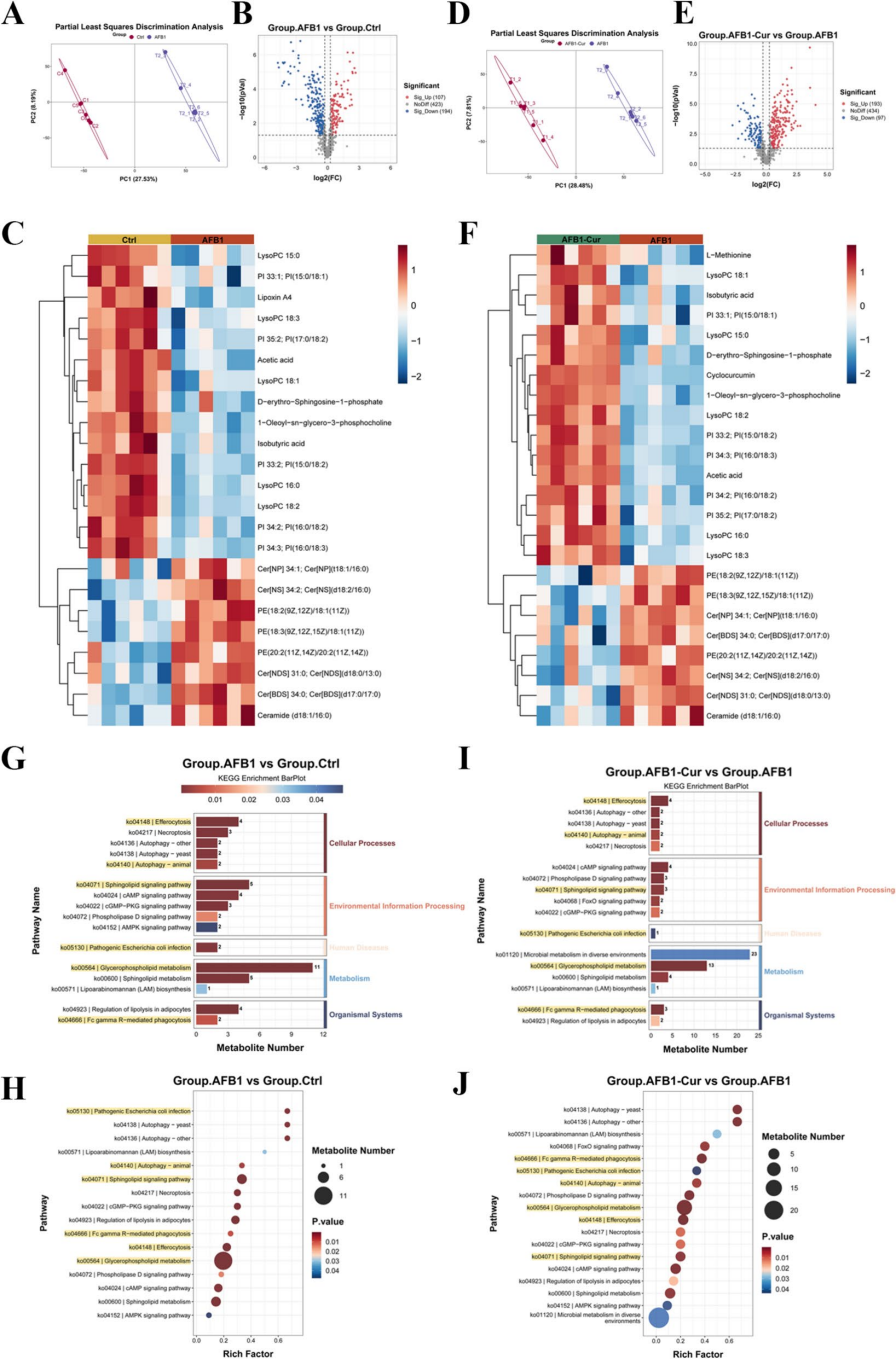
Effect of curcumin on AFB1-induced ruminal microbial metabolic disorders in sheep. **A** Partial least squares discriminating analysis (PLSDA) (Ctrl vs AFB1). **B**, **C** Volcano plot and heat map of differentially expressed metabolites (DEMs) (Ctrl vs AFB1). **D** PLSDA between AFB1 and AFB1-Cur groups. **E**, **F** Volcano plot and heat map of DEMs (AFB1 vs AFB1-Cur). **G**, **H** Bar graphs and Bubble Plot of KEGG enrichment analysis (Ctrl vs AFB1). **I**, **J** Bar graphs and Bubble Plot of KEGG enrichment analysis (AFB1 vs AFB1-Cur)

This study examined the impact of curcumin supplementation on AFB1-induced rumen microbial metabolic disorders via untargeted metabolomic analysis of ruminal fluid. The partial least squares-discriminant analysis (PLS-DA) revealed clear clustering patterns between the Ctrl and AFB1 groups (Fig. 3A), indicating significant metabolic differences. A similar result was obtained by PLS-DA of the AFB1 and AFB1-Cur groups, which showed marked metabolic divergence, further confirming the regulatory role of curcumin in counteracting AFB1-induced disturbances (Fig. 3D). The volcano plot showed significant metabolic disparities between the Ctrl and AFB1 groups (Fig. 3B, Supplemental Table S7). The analysis identified 107 significantly upregulated metabolites and 194 significantly downregulated metabolites (*P* < 0.05). A similar observation was made when the AFB1 and AFB1-Cur groups were compared, demonstrating distinct metabolic shifts, characterized by 193 significantly upregulated metabolites and 97 significantly downregulated metabolites (*P* < 0.05, Fig. 3E, Supplemental Table S8). These results underscore the modulatory effects of curcumin on AFB1-induced metabolic dysregulation. A comprehensive analysis of the differential metabolites between the groups revealed distinct metabolic alterations. Compared to the Ctrl group, the AFB1 group exhibited a decrease in lysophosphatidylcholines (LPCs), phosphatidylinositols, and D-erythro-sphingosine-1-phosphate, alongside an increase in ceramides and phosphatidylethanolamines, as visualized in the heatmap (Fig. 3C). Conversely, the AFB1-Cur group (Fig. 3F) exhibited a reversal of these AFB1-induced metabolic perturbations, resulting in the restoration of the expression patterns of the aforementioned lipid species. Furthermore, curcumin supplementation led to an upregulation of L-methionine and cyclocurcumin, underscoring its function in mitigating AFB1-driven metabolic dysregulation and enhancing key bioactive metabolites.

KEGG pathway enrichment analysis of differential metabolites, as depicted in the enrichment bar plots and enrichment factor plots (Fig. 3G and H, Supplemental Table S9), demonstrated that metabolites altered between the AFB1 and Ctrl groups were primarily enriched in pathways such as Pathogenic Escherichia coli infection, autophagy, sphingolipid signaling pathway, Fc gamma R-mediated phagocytosis, efferocytosis, and sphingolipid metabolism. A comparative analysis of the AFB1-Cur and AFB1 groups (Fig. 3I and J, Supplemental Table S10) revealed significant enrichment of differential metabolites in the same core pathways, including autophagy, Fc gamma R-mediated phagocytosis, Pathogenic Escherichia coli infection, efferocytosis, sphingolipid signaling pathway, and sphingolipid metabolism. These findings highlighted the role of curcumin in modulating these biological processes disrupted by AFB1.

### Correlation of ruminal microbiota and metabolites of sheep

**Fig. 4:**
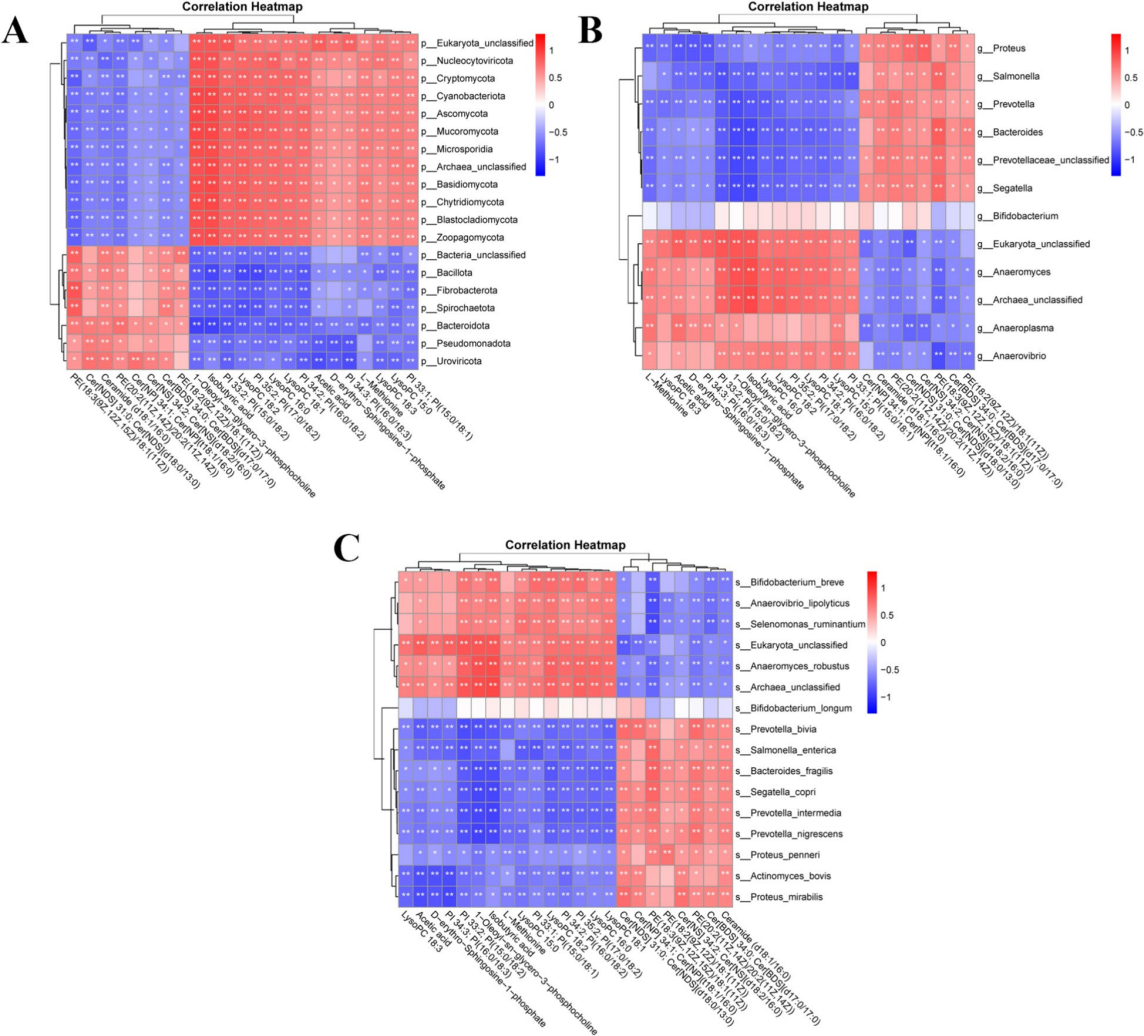
The correlation between ruminal metabolites and microbiota. **A**-, **B** and **C** The correlation between ruminal metabolites and microbiota at the phylum, genus, and species level, respectively (n = 6). * means *P* < 0.05, ** means *P* < 0.01

Based on spearman correlation analysis, the correlation between ruminal metabolites and microbiota at different taxonomic levels of sheep are shown in Fig. 4. At the phylum level (Fig. 4A), *Bacteroidota*, *Bacillota*, *Pseudomonadota*, and *Spirochaetota* showed significant positive correlations with ceramides and phosphatidylethanolamines (*P* < 0.05 or *P* < 0.01) but significant negative correlations with phosphatidylinositols and LPCs (*P* < 0.05 or *P* < 0.01). At the genus level (Fig. 4B), *Prevotella*, *Bacteroides*, *Prevotella_unclassified*, *Segatella*, *Proteus*, and *Salmonella* were significantly positively associated with ceramides and phosphatidylethanolamines (*P* < 0.05 or *P* < 0.01), while displaying significant negative correlations with phosphatidylinositols and LPCs (*P* < 0.05 or *P* < 0.01). Similarly, at the species level (Fig. 4C), *Segatella copri*, *Bacteroides fragilis*, *Prevotella intermedia*, *Proteus penneri*, and *Salmonella enterica* exhibited strong positive associations with ceramides and phosphatidylethanolamines (*P* < 0.05 or *P* < 0.01) but significant inverse relationships with phosphatidylinositols and lysophosphatidylcholines (*P* < 0.05 or *P* < 0.01).

### Effect of curcumin on the impairment of ruminal barrier function induced by aflatoxin B1

**Fig. 5:**
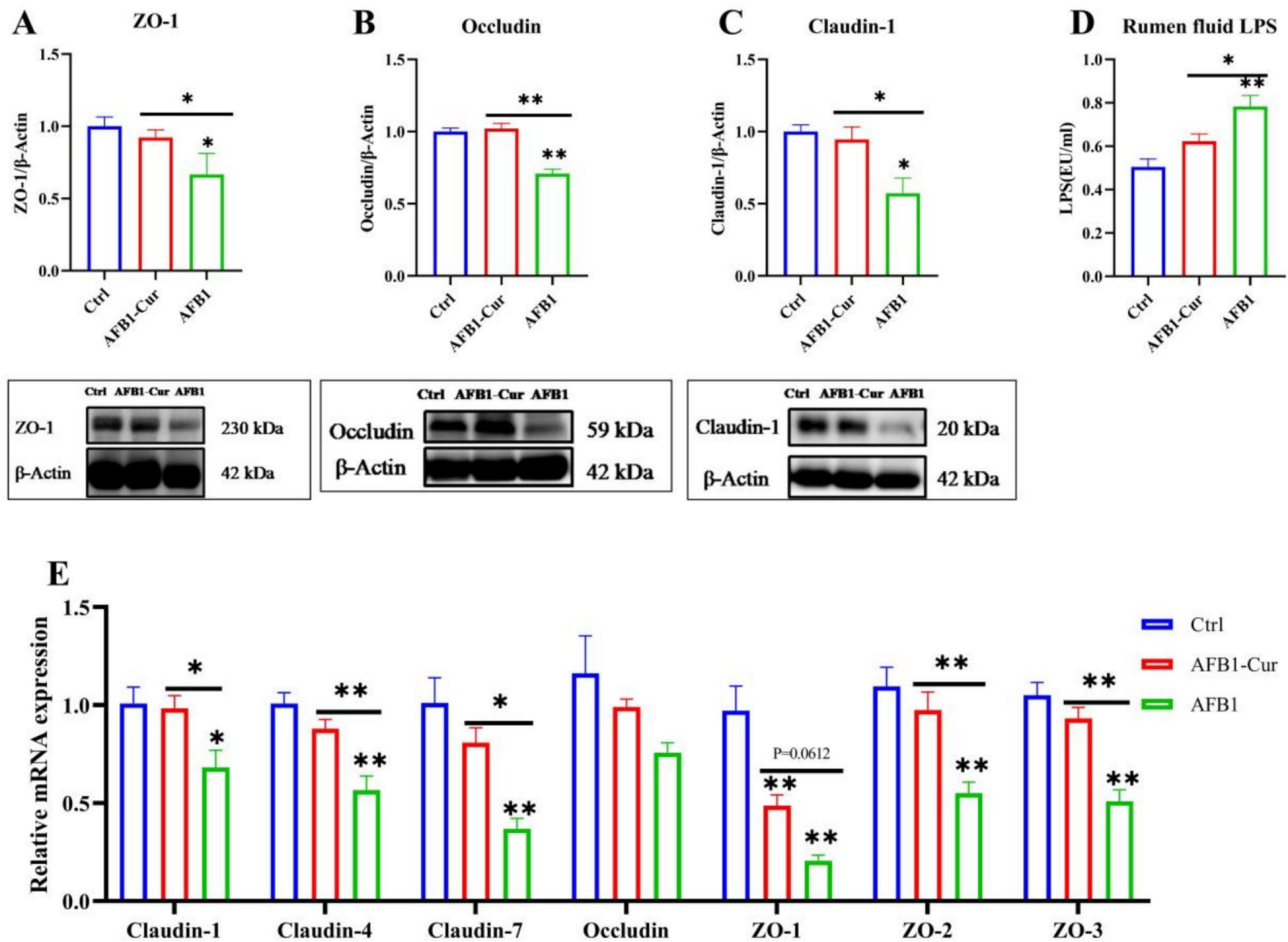
Effect of curcumin on the impairment of ruminal barrier function induced by AFB1. **A**, **B** and **C** Expression levels of the rumen barrier-related tight junctional protein ZO-1, Occludin, and Claudin-1 (n = 3). **D** LPS content in rumen fluid (n = 6). **E** Genes expression of the rumen barrier-related tight junctional proteins (n = 6). All data are expressed as mean ± SEM. * means *P* < 0.05, ** means *P* < 0.01

As shown in Fig. 5A-C, the AFB1 group exhibited significantly decreased protein expression levels of ZO-1, Occludin, and Claudin-1 compared to the Ctrl group (*P* < 0.05 or *P* < 0.01). Conversely, the AFB1-Cur group exhibited a substantial increase in the protein expression of ZO-1, Occludin, and Claudin-1 compared to the AFB1 group (*P* < 0.05 or *P* < 0.01), thereby underscoring curcumin's protective role in mitigating AFB1-induced disruption of rumen barrier integrity. As shown in Fig. 5D, rumen fluid LPS levels were significantly increased in the AFB1 group compared to the Ctrl group (*P* < 0.01). Conversely, the AFB1-Cur group exhibited a significant reduction in LPS content compared to the AFB1 group (*P* < 0.05), thereby demonstrating curcumin's efficacy in attenuating AFB1-induced endotoxin accumulation.

As demonstrated in Fig. 5E, in comparison with the Ctrl group, the AFB1 group demonstrated significant downregulation in the gene expression of Claudin-1, Claudin-4, Claudin-7, ZO-1, ZO-2, and ZO-3 (*P* < 0.05 or *P* < 0.01). In contrast, the AFB1-Cur group exhibited significant upregulation of Claudin-1, Claudin-4, Claudin-7, ZO-2, and ZO-3 (*P* < 0.05 or *P* < 0.01) compared with the AFB1 group. These findings highlighted the restorative effects of curcumin on rumen barrier integrity disrupted by AFB1 exposure.

### Effect of curcumin on liver injury induced by AFB1 in sheep

**Fig. 6:**
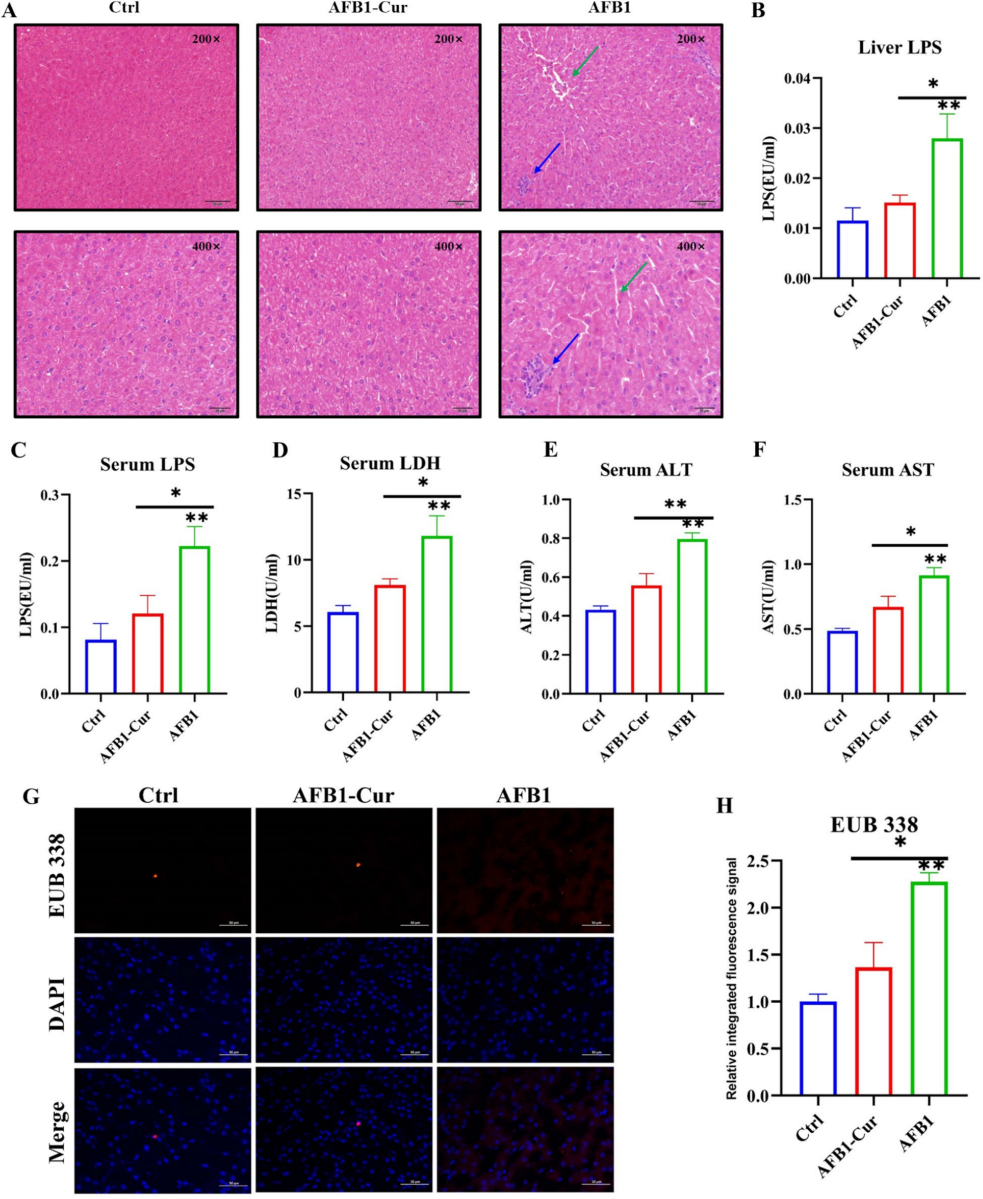
Effect of curcumin on liver injury induced by AFB1. **A** Histological examination of liver (200 × and 400 × , n = 3), green arrows indicate hepatic cord disruption, and blue arrows indicate inflammatory cell infiltration. **B**, **C** LPS content in liver and blood (n = 6). (D-F) The liver function indicators in serum (LDH, ALT, and AST; n = 6). **G**, **H** The fluorescence in situ hybridization (FISH) analysis of liver and the relative fluorescence intensity of EUB 338 probe (n = 3). All data are expressed as mean ± SEM. * means *P* < 0.05, ** means *P* < 0.01

To evaluate the impact of curcumin on AFB1-induced hepatic injury in sheep, hematoxylin and eosin (H&E)-stained liver tissue sections were examined under light microscopy at 200 × and 400 × magnification (Fig. 6A). The histopathological analysis revealed severe hepatic damage in the AFB1 group, characterized by hepatic cord disruption and inflammatory cell infiltration compared with the Ctrl and AFB1-Cur groups. To further evaluate hepatic injury, key biochemical indicators reflecting liver damage were measured in both liver and blood. As shown in Fig. 6B-F, the AFB1 group showed a marked increase in LPS levels of the liver and blood (*P* < 0.01), lactate dehydrogenase (LDH), ALT, and AST of blood (*P* < 0.01) compare with Ctrl group. In contrast, the AFB1-Cur group demonstrated significant reductions in hepatic LPS, LDH, ALT, AST, and LPS of blood compared to AFB1 group (*P* < 0.05 or *P* < 0.01), underscoring curcumin's hepatoprotective effects against AFB1-induced toxicity.

Given the observed dysbiosis of rumen microbiota, compromised rumen barrier function, and altered hepatic LPS levels, bacterial translocation to the liver was assessed using FISH. The EUB 338 probe (which is specific for bacterial 16S rRNA) labeled with Cy3 emitted red fluorescence, while blue fluorescence corresponded to nuclei counterstained with DAPI (Fig. 6G and H). A comparison of the AFB1 group and the Ctrl group revealed a marked increase in red fluorescence intensity (*P* < 0.01), suggesting a heightened degree of bacterial infiltration into hepatic tissue. Conversely, the AFB1-Cur group exhibited a significant reduction in fluorescence intensity compared to the AFB1 group (*P* < 0.05), demonstrating curcumin's efficacy in mitigating AFB1-driven bacterial translocation.

### Curcumin alleviated AFB1-induced hepatic pyroptosis through TLR4-NF-κB-NLRP3 signaling pathway

**Fig. 7:**
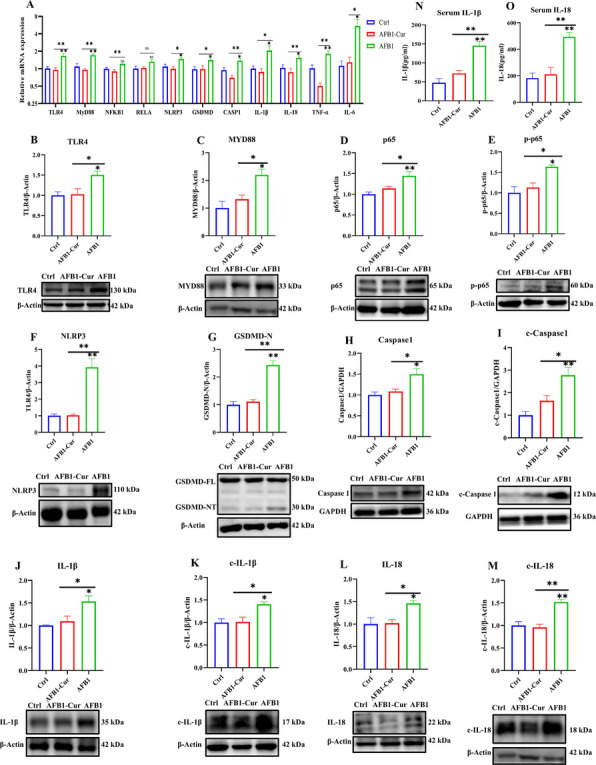
Curcumin alleviated AFB1-induced hepatic pyroptosis through TLR4-NF-κB-NLRP3 signaling pathway. (A) the relative mRNA expression of pyroptosis-related genes of liver (n = 6). **B**, **C**, **D**, **E** and **F**) the relative expression of TLR4 pathway-related protein (TLR4, MYD88, p65, p-p65, and NLRP3; n = 3). **G**-**I** the relative expression of hepatic pyroptosis-related protein (GSDMD, Caspase-1, and c-Caspase-1; n = 3). (J-M) the relative protein expression of the inflammatory cytokines (IL-1β, c-IL-1β, IL-18, and c-IL-18; n = 3). (N, O) The content of IL-1β and IL-18 in serum of sheep (n = 6). All data are expressed as mean ± SEM. * means *P* < 0.05, ** means *P* < 0.01

To elucidate the specific mechanisms by which curcumin alleviates AFB1-induced hepatic injury, the expression of genes and proteins associated with pyroptosis-related signaling pathways was analyzed. As shown in Fig. 7A, compared with the Ctrl group, the AFB1 group exhibited significantly increased relative mRNA expression levels of TLR4, MyD88, NFKB1, RELA, NLRP3, GSDMD, CASP1, IL-1β, IL-18, TNF-α, and IL-6 in liver (*P* < 0.05 or *P* < 0.01). Conversely, the AFB1-Cur group demonstrated significant downregulation of TLR4, MyD88, NFKB1, RELA, NLRP3, GSDMD, CASP1, IL-1β, IL-18, TNF-α, and IL-6 compared with the AFB1 group (*P* < 0.05 or *P* < 0.01), highlighting curcumin’s ability to suppress AFB1-driven activation of pyroptotic and inflammatory pathways in hepatocytes.

Subsequent Western blotting analysis (Fig. 7B-F) demonstrated that AFB1 treatment activated the TLR4-NF-κB-NLRP3 signaling pathway, with significantly elevated protein expression levels of TLR4, MYD88, p65, phosphorylated p65 (p-p65), and NLRP3 compared with the Ctrl group (*P* < 0.05 or *P* < 0.01). Conversely, the administration of AFB1-Cur inhibited the activation of this pathway. To further assess pyroptosis triggered by NLRP3 inflammasome assembly, pyroptosis-related proteins were analyzed (Fig. 7G-M). AFB1 treatment led to a significant upregulation in the expression of the cleaved N-terminal fragment of GSDMD (GSDMD-N), pro-Caspase-1, and cleaved Caspase-1 (c-Caspase-1), pro-IL-1β, cleaved IL-1β (c-IL-1β), pro-IL-18, and cleaved IL-18 (c-IL-18) compared with the Ctrl group (*P* < 0.05 or *P* < 0.01). Remarkably, AFB1-Cur treatment reversed these AFB1-induced changes in protein expression, highlighting curcumin's ability to counteract pyroptotic and inflammatory cascades.

Finally, serum levels of pyroptosis-related inflammatory cytokines were quantified using ELISA kits (Fig. 7N and 7O). Compared with the Ctrl group, AFB1 treatment significantly elevated serum IL-1β and IL-18 concentrations (*P* < 0.01). In contrast, relative to the AFB1 group, AFB1-Cur co-treatment markedly attenuated these AFB1-induced elevations in IL-1β and IL-18 levels (*P* < 0.01), thereby demonstrating the suppressive effect of curcumin on pyroptosis-associated inflammatory responses.

### Curcumin alleviates AFB1-induced suppression of autophagy in liver

**Fig. 8:**
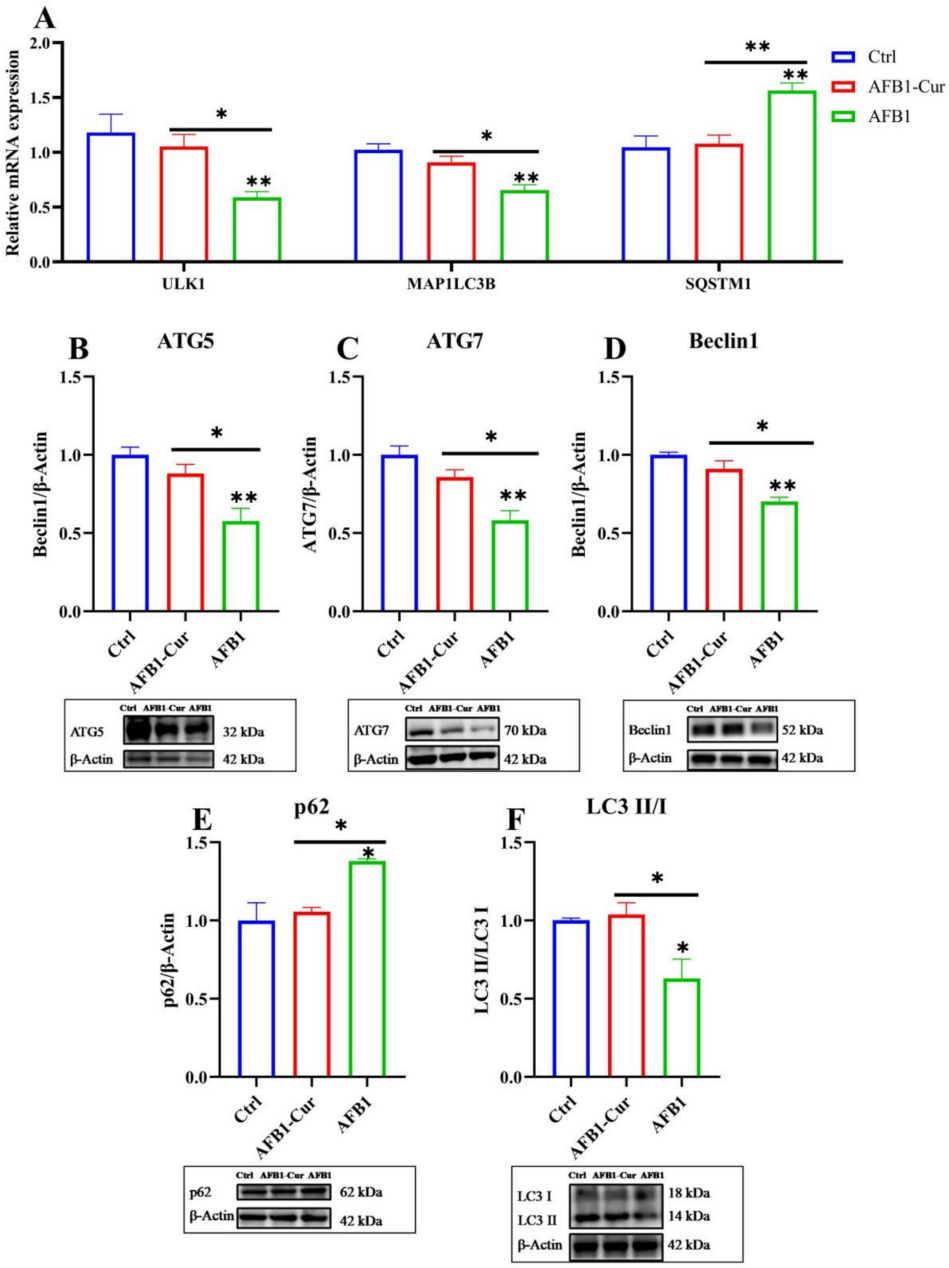
Curcumin alleviated AFB1-induced suppression of autophagy in liver. **A** the expression of hepatic autophagy-related genes (n = 6). (B-F) the expression of hepatic autophagy-related protein (ATG5, ATG7, Beclin1, p62 and LC-3; n = 3). All data are presented as mean ± SEM, * means *P* < 0.05, ** means *P* < 0.01

As shown in Fig. 8A, compared with the Ctrl group, AFB1 treatment significantly downregulated the expression of autophagy-related genes ULK1 and MAP1LC3B (*P* < 0.01), while upregulating the expression of SQSTM1 (*P* < 0.01). Remarkably, AFB1-Cur treatment markedly reversed these AFB1-induced alterations in gene expression compared to the AFB1 group, restoring levels of ULK1, MAP1LC3B, and SQSTM1 toward those observed in the Ctrl group (*P* < 0.05 or *P* < 0.01). As shown in Fig. 8B-F, compared with the Ctrl group, AFB1 treatment significantly downregulated the levels of autophagy-related proteins ATG5, ATG7, Beclin1, and LC3 II/I (*P* < 0.05 or *P* < 0.01), while markedly upregulating p62 protein expression (*P* < 0.05). In contrast, AFB1-Cur co-treatment effectively reversed these AFB1-induced protein expression alterations relative to the AFB1 group.

## Discussion

This study showed that AFB1 had no significant effect on the ADG, ADFI, F/G or liver weight of sheep. However, the supplementation of curcumin presented a moderate reduction in ADFI. Despite the lower feed intake, the AFB1-Cur group showed significant improvements in the primary endpoints of this study: namely, attenuated liver damage (e.g., reduced serum AST/ALT) and moderated changes in the gut microbiota profile. This indicated that curcumin's hepatoprotective and microbiota-modulating actions were effective even under conditions of reduced feed intake, suggesting these benefits were direct and not solely secondary to improved nutrition. Notably, previous study has reported that curcumin alleviates AFB1-induced growth retardation and renal index alterations in ducklings [29]. The absence of significant growth performance differences in our study may stem from the relatively short experimental duration, which AFB1 toxicity and curcumin's protective effects might not manifest in macroscopic growth metrics. The NH_3_-N reflects microbial nitrogen utilization and is a key substrate for microbial protein synthesis [30], while VFAs serve as the main energy source for ruminants [31]. In this study, AFB1 exposure led to a significant reduction in VFAs and NH_3_-N levels of ruminal fluid, whereas curcumin did not significantly increase their production compared with the AFB1 group. This may be because this study was short-term; after curcumin supplementation, the substantial amounts of VFAs produced in rumen were transported to other organs such as liver to alleviate AFB1-induced hepatic damage, rather than accumulating significantly in the rumen fluid.

A previous research has identified a robust correlation between rumen microbiota dysbiosis and various disease processes, especially hepatic injury [32]. It has been well-documented that toxic dietary components, including zearalenone and fumonisins, are known to disrupt rumen microbial homeostasis [33]. Conversely, dietary interventions, particularly phytogenic extracts like anthocyanins, citrus flavonoids, and curcumin, have been shown to restore microbiota balance [34–36]. To clarify the mechanism by which curcumin counteracts AFB₁-induced rumen microbial dysbiosis, this study conducted a multi-taxonomic analysis covering phylum, genus, and species levels. At the phylum level, curcumin effectively mitigated AFB₁-triggered compositional shifts, significantly reducing the elevated relative abundances of *Bacteroidota*, *Bacillota*, *Pseudomonadota*, and *Spirochaetota*. These findings are consistent with previous studies, where Lin et al. (2022) reported increased *Bacteroidota* and *Spirochaetota* abundances in AFB1-exposed Dorper rams [20] and Zhao et al. (2021) observed a similar upregulation of *Bacillota* in AFB1-administered mice [37]. These phyla have clear pathological relevance: *Pseudomonadota* is linked to necrotizing enterocolitis [38], pro-inflammatory *Bacteroidota* to inflammatory bowel disease [39], and enriched *Bacillota* to mild-to-moderate non-alcoholic fatty liver disease (NAFLD), underscoring the clinical significance of curcumin’s microbiota-modulating effects [40]. Further down to the genus level, AFB1 significantly increased the abundances of *Prevotella*, *Bacteroides*, and *Segatella*, while curcumin reversed these changes. *Prevotella*, a commensal-turned-opportunistic pathogen via LPS, contributes to periodontitis and colitis during dysbiosis [41]. *Bacteroides* may translocate extraintestinally and become pathogenic when the intestinal barrier is impaired [42], and Segatella is associated with gut inflammation[43]. Similar to present study, previous report has shown that high-dose curcumin reduced AFB1-induced elevation of *Bacteroides* abundance in ileum of duck This aligns with cross-species evidence showing curcumin reduced AFB₁-induced *Bacteroides* elevation in ducks and broiler chickens [29, 44]. At the species level, curcumin attenuated AFB₁-induced increases in pathogenic taxa (*Segatella copri*, *Bacteroides fragilis*, *Prevotella intermedia*, *Proteus penneri*, *Salmonella enterica*). *Segatella copri* links to liver cirrhosis and colorectal cancer [45]. *Bacteroides fragilis*, *Bacteroides fragilis* (a Gram-negative obligate anaerobe and opportunistic pathogen colonized the mucosal surfaces of ruminant digestive tracts) and *Prevotella intermedia* are inhibited by curcumin in vitro [46, 47], with the latter exacerbating immune dysregulation and polymicrobial infections [48]. *Proteus penneri* is a diarrheagenic pathogen [49], and *Salmonella enterica* invades the intestinal barrier to cause gastroenteritis [50]. Collectively, these multi-level findings confirmed curcumin's capacity to counteract AFB1-driven enrichment of pathogenic species, aligning with its documented antimicrobial and anti-inflammatory properties. The rumen microbiota diversity revealed that curcumin significantly mitigated AFB1-induced reductions in species richness (alpha diversity) and induced distinct clustering of treatment groups (beta diversity). Consistent with the present findings, studies have reported that AFB1 exposure decreases the Chao1 index of murine gut microbiota [51, 52], while curcumin counteracts this decline [53]. The observed alterations in rumen microbiota composition at the phylum, genus, and species levels are consistent with the hypothesis that curcumin effectively restores the disrupted microbial equilibrium in the rumen caused by AFB1.

Curcumin has been demonstrated to exert a substantial impact on the pathways that are enriched by differentially abundant microbial unigenes under the influence of AFB1 exposure. The upregulation of peptidoglycan biosynthesis, LPS biosynthesis, bacterial invasion of epithelial cells, and NOD-like receptor signaling pathways under AFB1 treatment indicates enhanced bacterial activity in the rumen, aligning with the observed increase in bacterial domain relative abundance. The reversal of these alterations by curcumin demonstrates its capacity to suppress bacterial overproliferation. Furthermore, the upregulation of *Pseudomonas aeruginosa* and *Escherichia coli* biofilm formation pathways under AFB1 suggests enhanced bacterial survival through protective biofilm assembly, which may contribute to persistent and treatment-resistant infections. Notably, curcumin counteracted this process, thereby attenuating biofilm-related virulence mechanisms.

Phosphatidylethanolamine (PE) constitutes 15%–25% of the total phospholipids in animal cell membranes [54]. Importantly, it also acts as both an essential membrane component and a critical cofactor for pathogenic organisms [55]. In NAFLD, altered hepatic lipid composition, particularly the phosphatidylcholine (PC)-to-PE ratio, is linked to steatosis and inflammation. Reduced ratio of PC and PE has been observed in liver biopsies of non-alcoholic steatohepatitis (NASH) patients [56]. This study found that curcumin significantly reduced AFB1‑induced elevation of PE levels, which were enriched in the pathogenic Escherichia coli infection pathway via KEGG analysis. Consistent with our findings, Zhou et al. (2021) observed increased PE and other pro‑inflammatory lipids in the feces of AFB1‑treated mice, indicating enhanced intestinal epithelial inflammation. [57]. The findings outlined here collectively underscore the potential of curcumin to mitigate AFB1-driven lipid dysregulation and the associated inflammatory cascades.

Curcumin has been shown to significantly attenuate the AFB1-induced increase in ceramide levels, which are primarily enriched in sphingolipid metabolism and signaling pathways. Ceramides, key mediators of sphingolipid metabolism, accumulate in tissues and plasma under conditions of metabolic dysfunction, dyslipidemia and inflammation; elevated plasma ceramide levels are established risk factors for cardiovascular events[58]. They stimulate NLRP3 inflammasome assembly to boost IL-1β production, upregulate TNF-α and IL-6 expression in adipocytes, and thus drive chronic inflammation [59], while also acting as viral entry receptors to facilitate viral replication site formation and increase infection susceptibility [60]. This phenomenon is consistent with the observed increase in rumen viral domain microbiota abundance. Furthermore, AFB1 altered sphingolipid profiles in brain of zebrafish, significantly upregulated ceramide level enriched in sphingolipid metabolism pathways [61]. Efferocytosis, the phagocytic clearance of apoptotic cells by both professional (macrophages/dendritic cells) and non-professional (epithelial/fibroblasts) phagocytes, is essential for resolving inflammation and maintaining tissue homeostasis [62]. Impairment of efferocytosis leads to the accumulation of dying cells, which can trigger a series of adverse outcomes, including autoimmunity, necrosis, and pathological inflammation [63, 64]. This process suppresses pro-inflammatory cytokines while promoting anti-inflammatory mediators to resolve inflammation [65]. LPCs has been identified as a crucial initiator of efferocytosis, functioning by recruiting phagocytes to sites of apoptosis through the action of chemoattractants [66]. Notably, studies have documented a depletion of PC and LPCs in the hepatic and circulatory systems of patients with NAFLD and NASH [67]. Wang et al. (2023) demonstrated that while immune microenvironment scores varied significantly only in HBV-induced end-stage liver disease (ESLD), broad dysregulation of inflammatory cytokines was observed across multiple etiologies of noncancerous ESLD, linking cytokine elevation to systemic immune microenvironment disturbance. These findings affirm that inflammatory cytokine profiles reflect broader immune microenvironment dysregulation and serve as biomarkers for systemic inflammatory and immune dysfunction [68]. The present study revealed that curcumin counteracted AFB1-induced reductions in LPC species, with differential LPCs enriched in efferocytosis pathways, indicating impaired efferocytic clearance of dying cells and ongoing inflammatory processes in rumen fluid.

The ruminal epithelium plays a pivotal role as an immune and biological barrier in ruminants, with its structural integrity being imperative for rumen health. Elevated levels of LPS, derived from the lysis of Gram-negative bacteria in the rumen, have been demonstrated to impair ruminal barrier function [69]. When the ruminal epithelium is compromised, opportunistic pathogens and microbial toxic metabolites, such as LPS, may translocate across the epithelial layer into the systemic circulation, thereby triggering systemic inflammatory responses [70]. In this study, dietary curcumin supplementation significantly mitigated AFB1-induced reduction in tight junction proteins (ZO-1, Occludin, and Claudin-1). Likewise, Pan et al. (2024) demonstrated that AFB1 significantly decreased expression of ZO-1, Occludin, and Claudin-1 at gene and protein levels in the ileal epithelium of ducks, whereas curcumin supplementation at varying doses markedly restored these tight junction components, effectively alleviating AFB1-induced intestinal epithelial damage [53]. Furthermore, our results revealed that curcumin significantly attenuated AFB1-driven increases in free LPS concentrations within rumen fluid. This reduction was consistent with metagenomic evidence indicating that curcumin ameliorated AFB1-induced ruminal microbiota dysbiosis, thereby suppressing the proliferation and lysis of Gram-negative bacteria responsible for LPS release. The observed positive correlation between LPS content and ruminal injury severity further corroborated the hypothesis that curcumin effectively mitigated both microbial dysregulation and barrier dysfunction in the rumen.

Given the significant alterations in ruminal free LPS levels following AFB1 and curcumin treatments, LPS concentration in serum was detected. The results demonstrated that curcumin markedly attenuated AFB1-induced elevation of serum LPS. Subsequent histopathological examination of liver demonstrated pronounced inflammatory cell infiltration in the AFB1 group, which was significantly alleviated by curcumin. Similarly, Wang et al. (2022) observed AFB1-induced hepatic injury in mice, characterized by pale cytoplasmic staining, hepatocyte edema, steatosis, and inflammatory cell infiltration, with high-dose curcumin mitigating these pathological changes [71]. The disruption of hepatocyte membrane integrity by AFB1 has been shown to lead to the leakage of intracellular enzymes, such as ALT, AST, and LDH, into the bloodstream, resulting in elevated serum enzymatic activities [72, 73]. This study found that AFB1 increased AST, ALT, and LDH content in serum of sheep, indicating hepatic cellular damage and liver injury. Curcumin supplementation led to a substantial decrease in the concentrations of these enzymes, suggesting its capacity to reverse AFB1-induced hepatotoxicity. These findings are consistent with a previous report that curcumin significantly reduced serum ALT and AST elevations induced by AFB1 in rats, thereby validating its hepatoprotective role against mycotoxin-induced liver damage [74]. Curcumin significantly attenuated AFB1-induced elevation of hepatic LPS levels, while bacterial FISH revealed markedly higher fluorescence intensity in the AFB1 group compared to the curcumin-supplemented group. Bacterial translocation, defined as the migration of gut bacteria and their endotoxins (e.g., bacterial lipopolysaccharides and peptidoglycans) from the gastrointestinal lumen to mesenteric lymph nodes and extraintestinal sites [75], was further implicated in AFB1-induced toxicity. In support of this mechanism, a prior work observed in AFB1-exposed mice not only mild hepatic inflammatory infiltration and elevated serum AST/ALT levels but also systemic accumulation of LPS in serum and liver tissues, accompanied by increased bacterial translocation to the liver [52]. Collectively, these findings demonstrated that AFB1 disrupts intestinal barrier integrity, facilitating LPS leakage and bacterial translocation, while curcumin supplementation potently reverses these adverse pathological changes.

The mammalian TLR family is comprised of 13 members, which are activated by pathogen-associated molecular patterns (PAMPs) such as structural motifs expressed by bacteria, fungi, and viruses [76]. Multiple PAMPs, including LPS from Gram-negative bacterial membranes, can activate TLR4 [77, 78]. Upon activation, TLR4 triggers the MyD88-dependent signaling pathway at the plasma membrane, where MyD88 oligomeric complexes initiate downstream NF-κB signaling cascades [79]. The activation of NF-κB has been shown to promote the expression of NLRP3, pro-IL-1β, and pro-IL-18. Subsequent to this, NLRP3 oligomerizes and binds to the adaptor protein ASC to form an inflammasome complex, which in turn recruits and activates caspase-1. The activated caspase-1 then cleaves pro-IL-1β and pro-IL-18 into their bioactive mature forms (IL-1β and IL-18), and processes GSDMD, a hallmark protein of pyroptosis [80]. The cleavage of GSDMD generates membrane pores, leading to pyroptotic cell death, while mature inflammatory cytokines such as IL-1β and IL-18 are released extracellularly, amplifying inflammatory responses [81]. This study revealed that curcumin significantly suppressed AFB1-induced activation of the TLR4/NF-κB signaling pathway, thereby inhibiting the assembly and maturation of the NLRP3 inflammasome and ultimately attenuating hepatic pyroptosis. Concurrently, serum levels of pyroptosis-associated inflammatory cytokines, IL-1β and IL-18, were markedly reduced, further corroborating that curcumin alleviated AFB1-triggered hepatocyte pyroptosis. Consistent with our findings, curcumin supplementation mitigated AFB1-induced hepatic pyroptosis and fibrosis in ducks via the JAK2/NLRP3 signaling pathway [82]. Notably, several studies have reported that curcumin alleviates AFB1‑induced pyroptotic liver injury and oxidative stress in mice [71], as well as AFB1‑mediated inflammatory damage and pyroptosis in the duck ileum [53]. Furthermore, prokineticins (PROK) and their G protein-coupled receptor (GPCR) systems are implicated in the regulation of inflammation, particularly within the gastrointestinal tract. These receptor systems may contribute to the modulation of inflammation in liver and intestinal diseases [83]. Future studies should explore whether and how microbial signals regulate PROK/PROKR expression and activity in gastrointestinal and hepatic tissues, potentially uncovering novel mechanisms in the pathogenesis of diseases driven by the rumen-liver axis. Hendrawan et al. (2024) demonstrated that nano‑curcumin formulation significantly enhanced bioavailability and tissue solubility, thereby improving its protective efficacy against stress‑induced placental and fetal developmental impairments in mice [84]. The administration of nano‑curcumin at optimized doses (21–24.5 mg/kg B.W.) effectively reduced VEGF expression and increased fetal length under noise‑stress conditions, highlighting the importance of formulation strategies in maximizing curcumin’s therapeutic potential [84]. These findings support the translational relevance of nano‑formulations in mitigating toxin‑ or stress‑related tissue injury through enhanced delivery and bioactivity. While curcumin exerts its hepatoprotective effects primarily through direct antioxidant activity and modulation of mitochondrial function, advanced bioengineering approaches such as the ROS-responsive and mitochondria-targeted vesicular system developed by Shen et al. (2025) demonstrate a more integrated strategy, combining targeted delivery, controlled release, and activation of mitophagy to simultaneously tackle oxidative stress and mitochondrial dysfunction in liver injury [85]. These findings underscore the broad protective potential of curcumin against AFB1 toxicity across species and tissues.

Autophagy, a cytoprotective mechanism, enhances cell survival under stress by recycling damaged cellular components, whereas its inhibition disrupts cellular homeostasis and induces apoptosis or necrosis[86]. This work revealed that AFB1 significantly suppresses the expression of autophagy-related genes ULK1 and MAP1LC3B, while upregulating SQSTM1 (encoding p62). These transcriptional alterations are markedly reversed by curcumin intervention. At the protein level, AFB1 downregulates key autophagy regulators, including ATG5, ATG7, Beclin1, and the LC3-II/LC3-I ratio, accompanied by p62 accumulation. However, these effects were counteracted by curcumin co-treatment. Autophagy-related proteins (ATGs) orchestrate multiple stages of autophagy, including initiation, elongation, maturation, and lysosomal fusion [87]. Specifically, ULK1 has been shown to drive the formation of the autophagosome via the assembly of the ULK1 complex. The Beclin-1/VPS34 complex has been identified as a facilitator of vesicle elongation, and ATG7 has been determined to be a mediator of the lipidation of LC3-I to LC3-II. Autophagy inhibition is characterized by a reduced LC3-II/LC3-I ratio and p62 accumulation due to impaired autophagic flux [88]. Sang et al. (2023) demonstrated that taraxasterol alleviates AFB1-induced autophagy suppression in broiler chicken liver by inhibiting the PI3K/AKT/mTOR pathway [14]. Autophagy has been shown to negatively regulate pyroptosis, and deficiencies in autophagy-related proteins (ATGs) have been observed to exacerbate pyroptotic cell lysis and IL-1β release [86]. Accumulating evidence indicates that autophagy suppresses pyroptosis through multiple mechanisms. Firstly, autophagy impedes pyroptosis by eradicating damage-associated molecular patterns (DAMPs) and PAMPs that activate inflammasomes [89]. Secondly, autophagy directly targets key components of pyroptotic signaling, such as inflammasome complexes and their downstream effectors (e.g., caspase-1), to inhibit pyroptosis initiation [90]. Moreover, autophagy modulates pyroptosis execution by downregulating the cleavage of GSDMD, a pore-forming protein essential for pyroptotic membrane permeabilization [91]. These findings underscore autophagy's role as a pivotal cellular safeguard against pyroptosis, operating through both indirect clearance of inflammatory stimuli and direct interference with pyroptosis-related molecular mechanisms.

## Conclusions

**Fig. 9:**
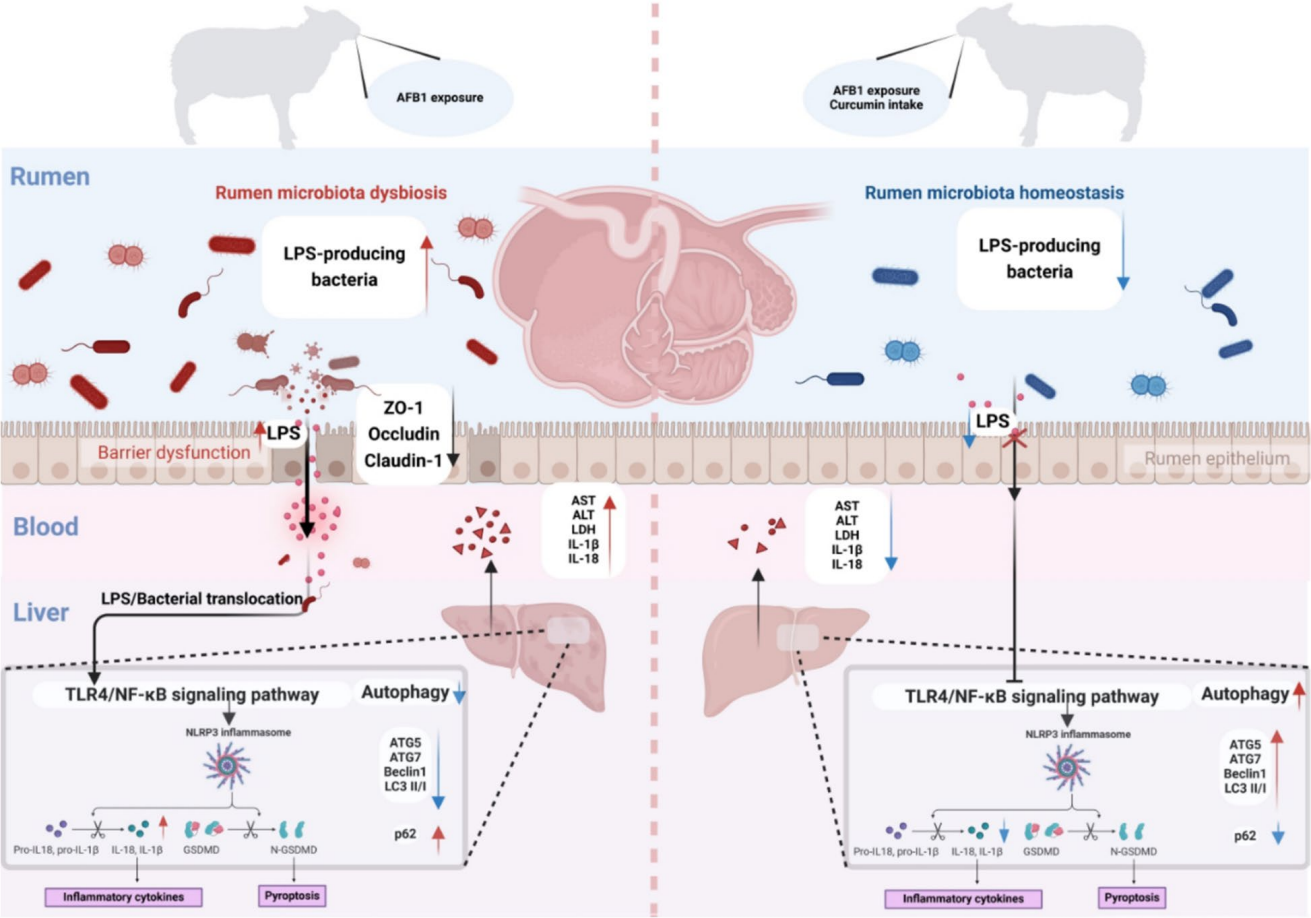
Mechanisms underlying the protective effect of curcumin against AFB1‑induced hepatic pyroptosis and autophagy inhibition in sheep. AFB1 exposure in the rumen induces dysbiosis of the ruminal microbiota, compromises the integrity of the rumen barrier, and facilitates the translocation of bacteria and their metabolites (e.g., lipopolysaccharide, LPS). This process triggers activation of the hepatic TLR4-NF-κB-NLRP3 signaling pathway, thereby promoting pyroptosis in hepatocytes. Co-administration of curcumin with AFB1 promotes the maintenance of ruminal microbiota homeostasis, preserves rumen barrier function, and reduces bacterial translocation. Collectively, these effects inhibit the activation of the TLR4-NF-κB-NLRP3 signaling pathway, thereby inhibiting hepatocyte pyroptosis. In addition, curcumin alleviates AFB1-induced inhibition of autophagy in hepatocytes

In summary, this work demonstrated that curcumin supplementation mitigated AFB1-induced disruption of the rumen microbiota-blood-liver axis, primarily through maintaining microbial and barrier homeostasis and dually modulating pyroptosis and autophagy in the liver (Fig. 9). These multifaceted mechanisms signify curcumin's promise as a dietary intervention for mycotoxin management. A limitation is the use of a single, high dose of curcumin under experimental conditions. Future prospects include investigating dose–response relationships, long-term safety, and the translatability of these benefits to commercial farming scenarios.

## Supplementary Information

Supplementary Material 1.

## Data Availability

The metagenome sequencing data obtained from the rumen fluid of sheep have been deposited in the NCBI Sequence Read Archive (SRA) database under the accession number (PRJNA1263030). The metabolomic data reported in this study have been deposited in the OMIX, China National Center for Bioinformation (https://ngdc.cncb.ac.cn/omix/release/OMIX010302) under the accession number (OMIX010302).
